# Nuclear and Mitochondrial Genome Characterization of a Clinical Isolate of the Emerging Pathogen *Wickerhamomyces anomalus*

**DOI:** 10.3390/jof12100753

**Published:** 2026-10-08

**Authors:** María José Cáceres-Valdiviezo, Luis Salvatierra-Chung Sang, Juan Carlos Tapia, Yessenia Acosta-Mosquera, Andrés Cáceres-Valdiviezo, Gabriel Morey-León, Juan Fernández-Cadena, Juan Ugalde, Derly Andrade-Molina

**Affiliations:** 1 Omics Sciences Laboratory, Faculty of Health Sciences, Universidad Espíritu Santo, Samborondón 092301, Ecuadorjtapian@unemi.edu.ec (J.C.T.);; 2 Facultad de Medicina, Universidad Nacional del Nordeste, Corrientes 1240, Argentina; 3 Human Development and Health Science Faculty, Universidad Ecotec, Km. 13.5 Samborondón, Samborondón 092302, Ecuador; 4 Center of Bioinformatics and Integrative Biology, Facultad de Ciencias de la Vida, Universidad Andres Bello, Santiago de Chile 837018, Chile; juan@ugalde.bio

**Keywords:** hybrid assembly, sequence annotation, yeast, mitochondrial genome, *Wickerhamomyces anomalus*, *Candida pelliculosa*, *Hansenula anomala*, clinical isolate

## Abstract

*Wickerhamomyces anomalus,* also known as *Candida pelliculosa,* has emerged as an opportunistic yeast implicated in invasive infections and hospital outbreaks, especially among neonatal and immunocompromised individuals. Despite its clinical relevance, the genomic determinants underlying the pathogenesis and virulence remain poorly understood. Here, we report the first high-quality nuclear and complete mitochondrial genome derived from a clinical isolate of *W. anomalus*. The nuclear genome spans 13.9 Mb with 34.5% GC content and contains 6305 protein-coding genes, 8235 coding sequences, and 147 tRNAs, with 98.1% completeness by BUSCO. The mitochondrial genome (47.6 kb) encodes 19 protein-coding genes, 26 tRNAs, two rRNAs, and exhibits atypical structural features, including inverted repeats and three LAGLIDADG (LD)-type homing endonuclease ORFs. We further assembled mitochondrial genomes from 12 additional clinical isolates and compared them with CpEC_UEES and publicly available *W. anomalus* mitochondrial genomes. Size, gene content and synteny were highly conserved, with structural variations largely limited to the LD ORFs. Phylogenetic analysis of 48 nuclear assemblies showed that clinical isolates are not monophyletic but are interspersed with environmental isolates across the tree. These assemblies provide the first comprehensive clinical genomic resources for *W. anomalus*, offering a reference framework for comparative and functional studies. The genomic innovations, together with a high content of repetitive and non-coding elements, suggest enhanced plasticity that may underpin metabolic flexibility and survival under host-associated stresses. These rearrangements represent an underappreciated mechanism of adaptation in emerging fungal pathogens, contributing to the clinical relevance of *W. anomalus.* These genomic resources facilitate rigorous comparative analyses to identify determinants of pathogenicity, virulence, and antifungal resistance, accelerating the development of improved diagnostics, surveillance, and management strategies for *W. anomalus* infections

## 1. Introduction

Fungal infections are responsible for more than 1.5 million deaths per year, with candidiasis the most common invasive fungal infections in hospitals [1,2,3]. Although *Candida albicans* remains the leading cause of invasive candidiasis, non-*albicans* yeasts are increasingly reported as causes of infections worldwide, surpassing *C. albicans* in some cases [1,4]. Among them, *Wickerhamomyces anomalus* (previously classified as *Pichia anomala*, *Hansenula anomala* and *Candida pelliculosa*) has emerged as an opportunistic pathogen associated with nosocomial outbreaks among neonates and immunocompromised patients [5]. These infections are linked to poor clinical outcomes, with reported mortality rates exceeding 40% in some cases [6,7,8], and clinical isolates are increasingly showing reduced susceptibility to azoles, including fluconazole [9,10].

*W. anomalus* is a diploid yeast with a broad ecological distribution, including plants, insects, soil, human tissues, and marine environments. Its wide metabolic and physiological diversity enables it to thrive over diverse environmental conditions, such as pH and extreme temperature fluctuations and osmotic stress [11,12,13]. These characteristics render *W. anomalus* a versatile organism, widely employed in the food, environmental, industrial, and medical sectors [14,15,16]. Metabolism and metabolic adaptation provide essential biosynthetic precursors and energy for critical biological functions in yeast [17], with mitochondria playing a crucial role in coordinating diverse anabolic and catabolic pathways fundamental to energy generation, metabolism, and pathogenesis [18]. In recent years, there has been a concentrated interest in the structural and functional characteristics of mitochondria in pathogenic yeasts such as *C. albicans* [19] and *C. auris* [20], while the mitochondrial genome of *W. anomalus* is unknown.

Research on the evolution and functionality of the mitochondrial genome can be challenging in certain fungal species, primarily due to notable difficulties associated with *de novo* assembly and intron prediction within the protein-coding genes (PCGs) [21,22,23]. Moreover, increasing evidence suggests that mitochondrial genetic features may contribute to the ability of specific yeasts to survive in hostile host environments, evade immune responses, and establish ongoing infections [24]. This knowledge gap is relevant in the case of *W. anomalus*, which despite its clinical relevance, the relationship between the genomic structure and its virulence and pathogenic potential have not been previously studied.

Intending to improve our knowledge on this opportunistic yeast, we present the first nuclear and complete mitochondrial genome assembly of a clinical isolate of *W. anomalus* using short and long reads generated by Illumina and PacBio sequencing technologies. We further assembled and compared the mitochondrial genomes of 12 additional clinical isolates from publicly available unassembled sequencing data and performed comparative and phylogenetic analyses. Our findings indicate that the genome of *W. anomalus* is highly congruent with available non-clinical genomes, while comparative mitochondrial genomics and phylogenetic analyses reveal that clinical isolates are not grouped into a distinct genomic lineage. Using *Candida* spp. and *Saccharomyces cerevisiae* as references, we identified a set of orthologous proteins such as synthetases, oligopeptide transporters, cell-wall-associated proteins, and antioxidant enzymes, which may be relevant to the virulence and pathogenic potential of *W. anomalus*. However, the majority of the genome remains uncharacterized.

Further studies are required to decipher the role of these hypothetical proteins in *W. anomalus*, as they might contribute to species-specific traits that could play a role in its pathogenesis.

## 2. Materials and Methods

### 2.1. Clinical Profile and Identification of W. anomalus

*W. anomalus* was isolated from the wound drainage of an 84-year-old woman during a one-year surveillance in 2019–2020, where some rare non-*Candida albicans* (NCA) were identified for the first time in Ecuador [25]. The isolate was identified using the VITEK 2 automated system, and culture strains were rearranged to a 2.0 McFarland standard (1.8–2.2; DensiCheck, BioMérieux, Marcy-l’Étoile, France) supplemented with 0.45% sterile NaCl following the manufacturer’s recommendations, and subsequently identified by sequencing of the rDNA internal transcribed spacer (ITS) using primers ITS1-ITS4 [26].

Given the increasing reports of azole-resistant clinical isolates of *W. anomalus* [9,10], antifungal susceptibility was determined with the VITEK^®^ 2 COMPACT using the AST-YS08 card (BioMérieux, Inc., Durham, NC, USA). It estimates the minimum inhibitory concentration (MIC), defined as the lowest antifungal concentration that inhibits visible fungal growth, for Amphotericin B, Caspofungin, Fluconazole, Micafungin, and Voriconazole. The isolate was deposited in the Mycotheque of the Laboratory of Omics Sciences, Universidad Espíritu Santo, Ecuador.

### 2.2. DNA Extraction, Genome Library, and Genome Sequencing

The genomic DNA of *W. anomalus* was extracted using the PureLink Genomic DNA Mini Kit (Invitrogen, Carlsbad, CA, USA) as instructed by the manufacturer with minor modifications. Briefly, a single colony from a freshly streaked YPD broth medium was inoculated into 5 mL and grown at 37 °C overnight with shaking at 200 rpm. Cell pellets were resuspended in the buffer protoplast (1 M sorbitol, 1% β-mercaptoethanol, 0.25 mg of lysozyme (Sigma-Aldrich, St. Louis, MO, USA), and 0.25 mg of lyticase (Sigma-Aldrich) and incubated at −80 °C overnight. Subsequently, a digestion buffer, proteinase K, and RNase A were added. The sample was homogenized by bead beating on a Bead Ruptor (Omni International^TM^, Kennesaw, GA, USA) for 5 min, repeated three times. Then, the lysis was mixed with a binding buffer and ethanol, and subsequently loaded into the purification spin column. The impurities were eliminated using a wash buffer, and the DNA was eluted using the elution buffer. The purified genomic DNA (gDNA) samples were stored at −30 °C until needed.

gDNA was then sent to Novogene Biotech Co., Ltd. (Beijing, China) for PacBio and Illumina sequencing. For long-read sequencing, HiFi SMRTbell libraries were prepared with the SMRTbell Express Template Prep Kit 2.0 (Pacific Biosciences, Menlo Park, CA, USA). The genomic DNA was fragmented, repaired, and A-tailed. SMRTbell hairpin adapters were then ligated to these prepared ends, and the AMPure PB beads (Pacific Biosciences, Menlo Park, CA, USA) were employed for the library’s concentration and purification steps. For sequencing, the library was checked for quantification and size distribution by a Qubit and bioanalyzer, respectively. Finally, the library was pooled, and the sequencing process was conducted using the PacBio Sequel II platform. Reads were trimmed, and high-quality regions were conserved. A total of 3.03 Gb was obtained, with a read N50 of 173.928 bp.

For short reads, the libraries and quality control were generated using a total amount of 1.0 μg DNA. Sequencing libraries were generated using NEBNext^®^ DNA Library Prep Kit (Illumina^®^, Ipswich, MA, USA) following the manufacturer’s recommendations. The genomic DNA was randomly fragmented to a size of 350 bp by shearing, then the DNA fragments were end polished, A-tailed, and ligated with the NEBNext adapter for Illumina sequencing, and further PCR enriched by P5 and indexed P7 oligos. The PCR products were purified (AMPure XP system), and the resulting libraries were analysed for size distribution by an Agilent 2100 Bioanalyzer (Agilent Technologies, Santa Clara, CA, USA) and quantified using real-time PCR. For sequencing, the qualified libraries are fed into Illumina sequencers after pooling according to their effective concentration and expected data volume. The DNA was sequenced using the Illumina NovaSeq 6000 platform, generating 9.1 Gb paired-end reads (150 bp each) with 93.38% Q30. Read quality was assessed using FastQC (v0.12.0), and adapter sequences and low-quality bases were removed by quality-filtering and trimming with Trimmomatic (v0.39) [27].

### 2.3. Genome Assembly

Highly accurate single-molecule consensus (CCS) reads were obtained by alignment of the subreads using the pbccs tool. Then, the CCS reads were used for genome assembly using different strategies. The first approach used only the generated long reads, using two different assemblers, SMARTdenovo (v1.0.0) [28] and Flye (v2.9.4) [29]. A second approach consisted of using the hybrid assemblers MaSuRCA (v4.1.2) [30] and Wengan (v0.2) [31] with default parameters. A fifth genome assembly, denominated as CANU-SMARTdenovo assembly, was built by adding a correction step of long reads with CANU [32] using the LRSDAY (v1.7.2) pipeline [33].

The set of contigs produced by each assembly strategy were corrected three consecutive times with the Pilon software (v1.24) [34] using the quality-filtered Illumina paired-end reads. The most contiguous assembly was then scaffolded with RagTag (v2.1.0) [35], using the reference genome GCA_019321675.1.

Assembly Assessment. Several quality metrics were collected to assess the quality of the genome assembly. Assembly contiguity was assessed using QUAST (v5.3.0) [36] and included a total number of contigs, total sequence, and contig NG50, among others. Reference-based analysis was performed using the published reference genome GCA_019321675.1.

The completeness of the assembly was measured using the BUSCO (v4) tool [37] with the Saccharomycetes database (saccharomycetes_odb10). Key assembly metrics, including N50, L50, longest contigs, number of contigs, GC content, and BUSCO scores, have been represented as SnailPlots using BlobToolKit (v4.3.0) [38].

Assembly QV was determined using Meryl (v1.4.1) and Merqury (v1.3) [39]. The Meryl databases were generated for each read file (PacBio long reads and Illumina paired-end reads) with a k-mer size of 17. Merqury was then run based on each Meryl database to determine the assembly QV.

### 2.4. Genome Annotation

The genome was annotated using an adapted MAKER-based pipeline in the LRSDAY pipeline [33,40]. Two different ab initio gene predictors were used: SNAP (v2013_11_29) [41] and Augustus (v3.5.0) [42,43], with soft masking in the masking repeating step. Augustus was trained using a *S. cerevisiae* model. Additionally, available protein sequences of *W. anomalus*, *C. glabrata*, *S. cerevisiae* (strain S288c), and *C. kefyr* were provided as homology evidence. A similar approach was performed to annotate the reference genomes: GCA_019321675.1 and GCA_001661255.1. tRNA genes were annotated using tRNAscan-SE [44], and mobile elements were predicted based on the predetermined TE-library available at LRSDAY. Annotation quality and completeness were assessed by BUSCO. Functional annotation was performed by mapping the predicted protein sequences to annotated orthologous groups using eggNOG-mapper v2 [45,46] and BlastKOALA (v3.1) [47].

### 2.5. Mitochondrial Genome Assembly, Annotation, and Comparative Analysis

The mitochondrial genome of CpEC_UEES was identified based on strong protein homology to the mitochondrial genes of *Saccharomyces cerevisiae* (strain S288c), *C. kefyr* (strain DMKU3-1042), and *C. glabrata* (strain ATCC 2001). This contig, along with contigs scf7180000000191 and scf7180000000194, were used as a template onto which the PacBio CCS reads were mapped using minimap2 v2.26 [48]. We then performed an independent assembly of the mapped long reads using CANU (v2.3), which resulted in a circularizable contig corresponding to the mitochondrial genome and another collapsed contig. The assembly was then corrected using Illumina short reads (as described in Section 3).

MitoHifi (v3.2.2) [49] was used to identify the mitochondrial sequence and circularize it. This was followed by annotation with MITOS2 (v2.1.10) [50] and MFannot (web portal) [51], and intron identification using RNAweasel (web portal) [52] and Infernal v1.1.5 [53]. Annotation inconsistencies were manually verified using BLAST [54]. Repeats were identified using RepeatMasker (v4.2.3) [55] and equicktandem (vEMBOSS:6.6.0.0) [56].

To expand the comparative dataset, raw sequencing data for 12 additional clinical isolates of *W. anomalus* were retrieved from NCBI. Reads were corrected using fastp (v1.3.6) [57] and later assembled *de novo* following the procedure described in [58]. Briefly, *de novo* assembly was performed with Spades (v3.13.1) [59] with k-mers 21, 33, 55, 77, 99 and 127, followed by mitochondrial identification and circularization with GetOrganelle (v1.7.7.1) [60] with default fungal mitochondrial parameters.

For comparative mitochondrial genome analysis, the 13 clinical mitochondrial genomes (CpEC_UEES + 12 isolates) and the publicly available sequences OR704056, OR704057, and OM055648 were compared using BRIG (v0.95) [61] with CpEC_UEES as the reference. Sequence identity thresholds were set at 70–100%.

### 2.6. Identification of Genes Related to Virulence/Drug Resistance

The PHI-base (v4.17) (Pathogen–host-interactions database) [62] and MaRDy (Mycology Antifungal Resistance Database) (v2.0) [63] databases were used for the identification of virulence and resistance genes, respectively. A BLASTP analysis was performed with an E-value cut-off of 1 × 10^−6^ using the predicted protein sequences as queries against the databases. Because a low E-value alone may result from a single shared domain, hits were further filtered by percent identity and query coverage. Only hits from *Candida* spp. and with a percent identity and query coverage greater than 60% and 80%, respectively, were considered. The identity threshold was chosen because the accuracy of functional annotation drops below 90% at sequence identities lower than approximately 60% [64]. For further confirmation, the hits were compared against the genes identified by the eggNOG-mapper.

### 2.7. Identification of Adhesin-Like Protein Sequences

We used multiple strategies to identify adhesin-related sequence structures. First, a preliminary prediction for a signal peptide was performed with the SignalP 6.0 server [65]. The organism group was set to Eukarya. We then combined these results with predictions for a C-terminal Glycosylphosphatidylinositol (GPI) anchor sequence. GPI-anchored proteins were identified using the PredGPI predictor (v1.0 web portal) [66]. Lastly, these sequences were input into FungalRV [67], a Machine learning fungal adhesion predictor trained with known adhesin protein sequences from 8 human fungal pathogens. Proteins with a factor score greater than zero were considered positive hits. Proteins that passed these filters were subjected to blastp against the non-redundant protein database limited to *Candida* (taxid: 5475), *Wickerhamomyces* (taxid: 599737), and *Cyberlindnera fabianii* (taxid: 36022). When no results were obtained, a general blastp was performed.

### 2.8. Phylogenetics Analysis

A total of 48 *W. anomalus* genome assemblies were used for phylogenetic analysis, comprising 36 assemblies available in NCBI (including CpEC_UEES), excluding those with a quality warning or unclear organism classification based on average nucleotide identity (ANI), and the 12 clinical isolates assembled in this study. The *Saccharomyces cerevisiae* S288C reference genome (GCF_000146045) was included as an outgroup, for a total of 49 assemblies. The UFCG (universal fungal core genes) profile (v1.0.6) [68] was applied to identify universal core protein sequences directly from genome assemblies using default settings. Phylogenetic tree reconstruction was then performed with the UFCG tree module from a concatenated alignment of 55 single-copy core marker genes and a maximum-likelihood tree was inferred with IQ-TREE under the JTT + F + I + G substitution model, with branch support assessed by 1000 ultrafast bootstrap replicates. The resulting tree was rooted with *S.cerevisiae* and visualized using Interactive Tree of Life (iTOL) [69].

### 2.9. Data Visualisation

Figures were constructed in R version 4.2.2 for Mac using the packages ggplot2 [70] and ggbreak [71]. Additionally, pyGenomeviz (v1.7.0) and pyCirclize (v1.10.1) were used to visualise genome alignments and the mitochondrial genome.

## 3. Results

### 3.1. Isolate Resistance Profile

The in vitro antifungal susceptibility test showed sensitivity to fluconazole, voriconazole, caspofungin, micafungin, amphotericin B, and flucytosine due to the low off–scale MIC value of Vitek 2 AST YS08 (Supplementary Table S1).

### 3.2. Assembling the Nuclear W. anomalus Genome

A combination of long-read PacBio and short-read Illumina sequencing was employed to obtain the genome of the *W. anomalus* strain isolated from a clinical sample (hereafter CpEC_UEES). Before assembly, the long PacBio subreads were pre-processed to generate Highly Accurate Single-Molecule Consensus reads (ccs). Different genome assembly algorithms were employed to create high-quality and high-coverage assemblies. The sequencing reads were assembled using the hybrid assemblers Wengan [31] and MaSuRCA [30], as well as the long-read assemblers Flye [29] and SMARTdenovo [28], with an additional CANU-corrected SMARTdenovo assembly (Canu-SMARTdenovo). The resulting assemblies were polished thrice with the short Illumina reads to remove sequence and structural errors. Traditional assembly metrics such as contig count, maximum contig size, N50, N75, genome size, consensus accuracy (QV), and BUSCO completeness score were used to compare the resulting assemblies (Table 1). Almost all assembly strategies yielded similar genome sizes, except Wengan, which produced the largest assembly (15.6 Mb), but was also the most fragmented (342 contigs) and had the lowest BUSCO completeness score (67%). The long-read assemblers produced highly contiguous assemblies, with Flye yielding the highest N50 (1.42 Mb) and SMARTdenovo and Canu-SMARTdenovo the largest contigs (3.63 Mb). However, their consensus accuracy was lower (QV 29.0–35.5). Read correction with CANU improved both the accuracy (QV from 29.0 to 35.3) and completeness (BUSCO from 88.7% to 96.6%) of the SMARTdenovo assembly without changing its contiguity.

Of all the assemblies, the CpEC_UEES assembly generated by MaSuRCA had the lowest number of contigs [20], the highest N75 (1.11 Mb), the highest consensus accuracy (QV 47.95 and 47.42 with short and long reads, respectively), and the highest BUSCO completeness score (98.1%). Therefore, this assembly was selected for downstream analysis.

The MaSuRCA contig set was then scaffolded using RagTag [29] and the previously published chromosome-level genome (GCA_019321675.1) as reference. Three contigs (scf7180000000186, scf7180000000191, scf7180000000194) comprising ~90 kb did not align to the reference genome and were further processed (Supplementary Figure S1). The final CpEC_UEES draft assembly yielded 10 scaffolds, with an N50 of 2,125,767 bp and a total length of 13,959,047 bp. Moreover, out of the 10 scaffolds, seven correspond to Chromosomes 1–7, and one to the mitochondrial genome.

The completeness of the genome assembly was assessed using the BUSCO tool [37], which evaluates the presence of universally conserved single-copy genes. BUSCO identified 2098 out of 2137 genes (98.1%) of the lineage-specific saccharomycetes group in the CpEC_UEES assembly as complete, with an additional fragmented gene. These results are comparable to those of the available assemblies of *W. anomalus* at NCBI. Additionally, more than 98% of PacBio and Illumina data were mapped to the final assembly with coverage of an average depth of 190X and 95X, respectively (Supplementary Table S2).

**Figure 1:**
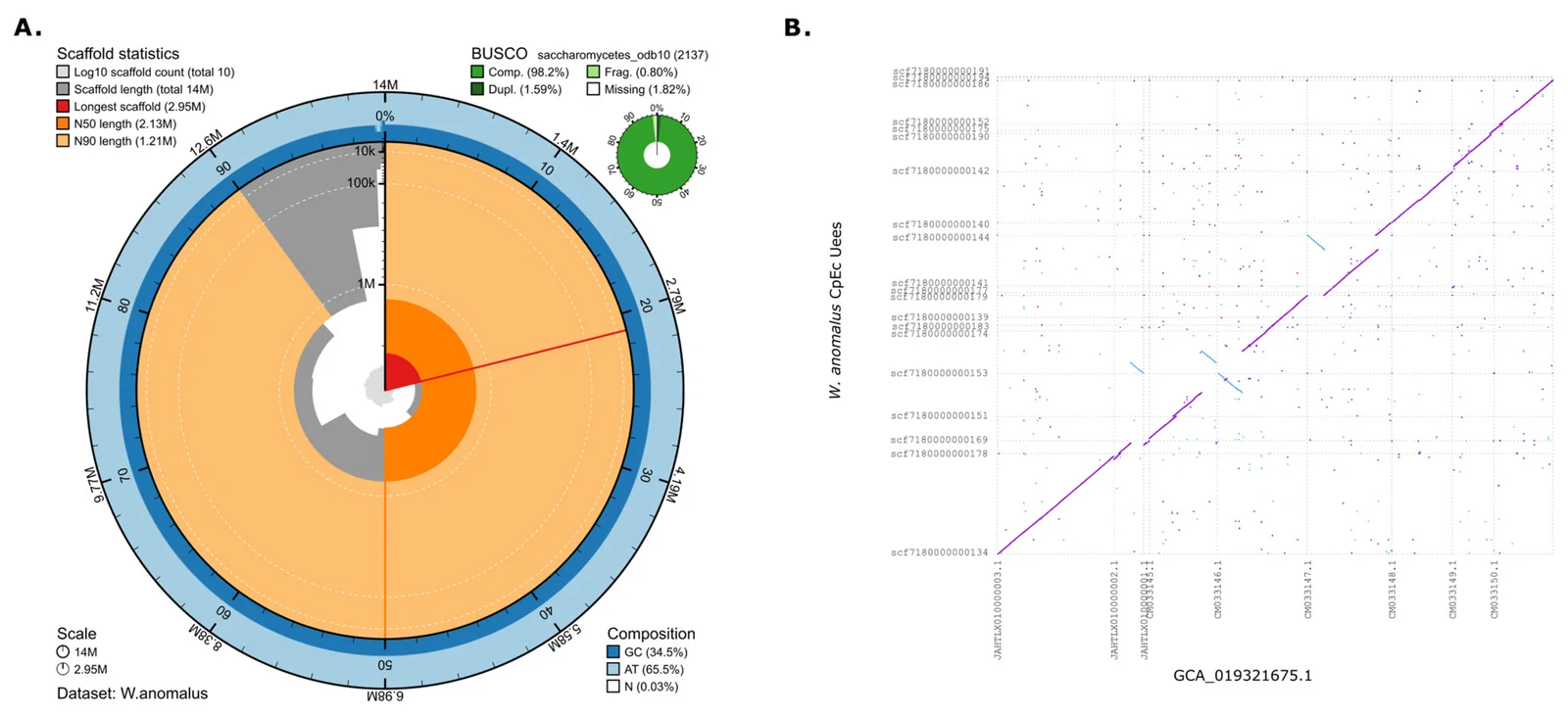
Genome assembly statistics and a dot-plot depicting the scaffold-level and contig-level assemblies, respectively, compared with the reference genome GCA_019321675.1. (**A**). Circular plot visualising the assembly metrics of the final *W. anomalus* draft genome assembly using BlobToolKit viewer [38]. (**B**). The dot-plot was generated using MUMmer 4.0.0 (NUCmer) [72]. Diagonal lines represent alignment between the reference genome (GCA_019321675.1) (horizontal) and CpEC_UEES (contigs, vertical). Purple lines indicate high similarity between strands, while light blue lines represent inverse alignment. Points near the diagonal indicate the co-linearity of genome sequences.

To evaluate the structural rearrangement of the CpEC_UEES genome, we aligned the draft assemblies from MaSuRCa and Canu-SMARTdenovo against the GCA_019321675.1 reference genome (Supplementary Figure S1). A significant macrosynteny and high sequence homology (>95% sequence identity) were observed. Although the MaSuRCa assembly contained 10 strand matches (purple lines) and four strand inversions (light blue lines) (Figure 1B), only two inversions were also present in the Canu-SMARTdenovo assembly (Supplementary Figure S1). Overall, these findings reveal that the structural rearrangement of the CpEC_UEES genome is largely conserved in comparison with the reference genome.

### 3.3. CpEC_UEES Genome Annotation

Protein-coding genes were annotated using a *de novo* homology-based approach. A total of 6324 putative genes were predicted, of which 6305 are nuclear encoded. Of the 6324 predicted genes, 5305 genes (84%) were functionally annotated by eggNOG-mapper [45]. The draft assembly was also used to identify 173 tRNAs (147 nuclear-encoded and 26 mitochondrial-encoded) and 11 truncated transposable elements belonging to the Ty1, Ty3, and Ty5 transposon families.

**Figure 2:**
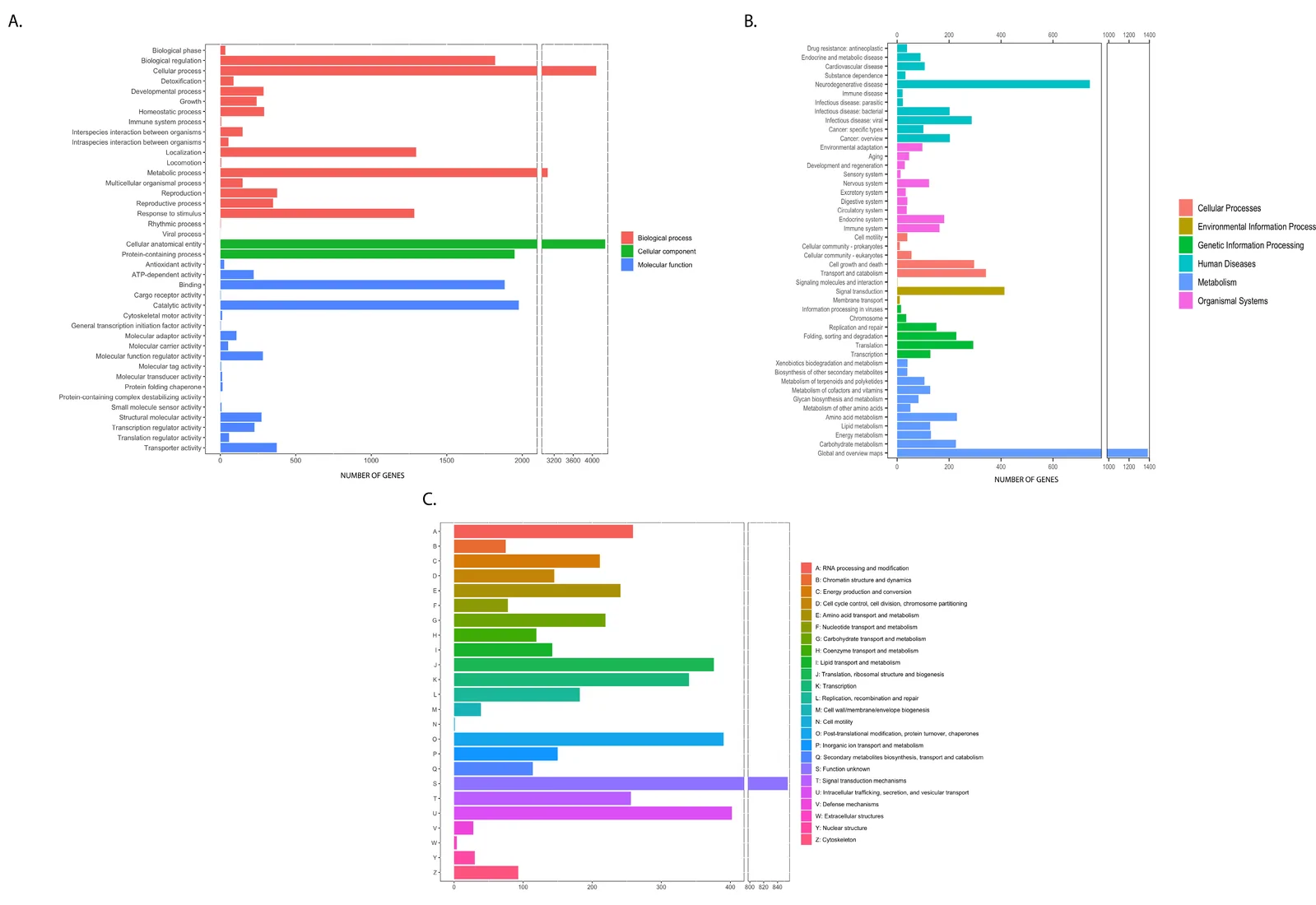
Distribution of Gene Ontology (GO), euKaryotic Orthologous Groups (KOGs), and Kyoto Encyclopaedia of Genes and Genomes (KEGG) annotations of predicted proteins from *W. anomalus* CpEC genome. (**A**). Functional distribution of the predicted proteins according to three major hierarchical GO terms: Biological process (red), Cellular component (green), Molecular function (blue). (**B**). Distribution of KOG annotation profiles. (**C**). Classification and functional distribution of the predicted proteins according to six major hierarchical KEGG terms.

From the 6324 predicted proteins, 4451 sequences (~70.4%) were annotated with GO terms and placed in three main domains: Molecular Function, Cellular Component, and Biological Process (Figure 2A, Supplementary Table S3). Notably, a substantial number of genes were related to cellular anatomical entities (4266 sequences) and Cellular processes (4083 sequences) within the Cellular component and Biological process domains, respectively. The Molecular function domain revealed a predominant presence of genes associated with catalytic and ligand-binding activity.

In parallel, 5197 genes were classified into KOG (euKaryotic Orthologous Groups) families across 24 categories. The most abundant classes were U (Intracellular trafficking and secretion), J (Translation ribosomal, structure, and biogenesis), O (Post-translational modifications, protein turnover, chaperones), and K (Transcription) (Figure 2B, Supplementary Table S4). Furthermore, KEGG annotation classified 3156 protein-coding sequences (49.9%) into 396 pathways (Figure 2C, Supplementary Table S5). Among these, the metabolic pathways (KO:01100) were the most enriched KEGG term with 638 genes, followed by biosynthesis of secondary metabolites (KO:01110, 261 genes), amyotrophic lateral sclerosis (KO:05014, 132 genes), and pathways of neurodegeneration-multiple diseases (KO:05022, 126 genes).

### 3.4. Mitochondrial Genome Assembly

**Figure 3:**
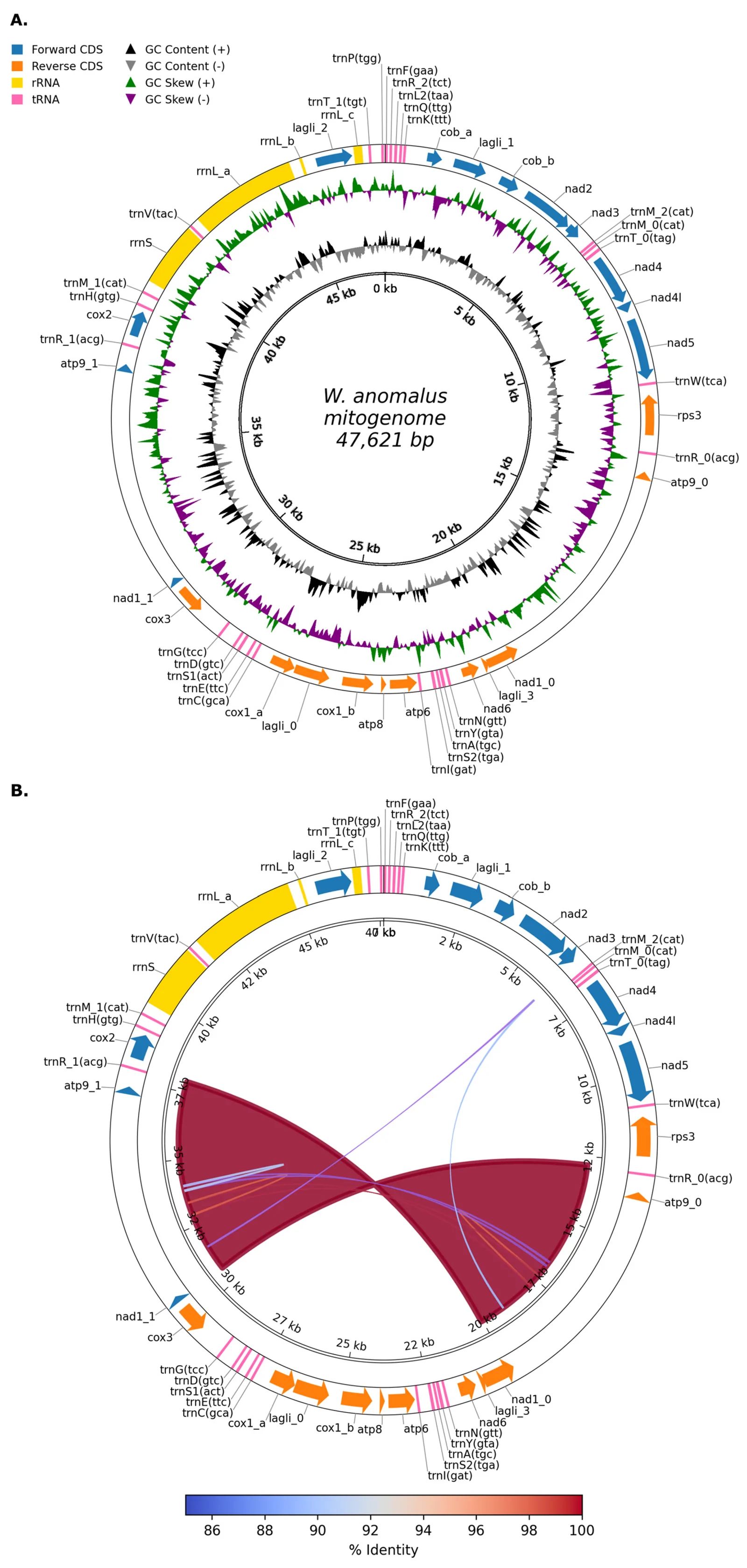
Circular representation of *W. anomalus* mitochondrial genome. (**A**). The outer track depicts annotated genomic features, including 16 core mitochondrial protein-coding genes (*atp6*, *atp8*, *atp9*, *nad1–nad6*, *nad4l*, *cob*, *cox1–cox3*, and *rps3*), 26 tRNA genes, the ribosomal RNA subunits (*rrnS* and *rrnL*), and three LAGLIDADG (LD)-containing endonuclease genes. Track 2 and Track 3 show GC content and GC skew, respectively. (**B**). Self-alignment of the mitochondrial genome, showing regions with e-value < 1 × 10^−10^ and sequence identity > 85%. Two ~7 kb regions exhibit 100% identity, corresponding to inverted repeats that include *atp9* and *trnR*.

Of the three unaligned contigs, we identified a single contig (scf7180000000186) corresponding to the mitochondrial genome based on strong homology to mitochondrial genes of *S. cerevisiae* (RefSeq: GCF_000146045.2), *N. glabratus* (RefSeq: GCF_010111755.1), and *K. marxianus* (RefSeq: GCF_001417885.1). The final mitochondrial genome assembly of *W. anomalus* consists of a single circular contig of 47,621 bp (GenBank ID: JBHVOA000000000) and a low GC content of 20.8%. It encodes 19 protein-encoding genes (PCGs), 26 tRNAs, and two rRNAs (Figure 3). Among these 47 genes, 27 are encoded by the leading strand, while the remaining 20 genes, including nine protein-coding genes (*atp6*, *atp8*, *atp9*, *cox1*, *cox3*, *nad1*, *nad6*, *rps3*, and *lagli*), and 11 tRNAs (*trnA*, *trnC*, *trnD*, *trnE*, *trnG*, *trnI*, *trnN*, *trnR*, *trnS1*, *trnS2*, and *trnY*) are encoded by the lagging strand (Figure 3). The genome contains three group I introns (two of which belong to the subtypes IA3 and ID) totalling 4634 bp (9.73% of the genome), of which 3039 bp (65.58%) correspond to LADLIDADG (LD) ORFs (open reading frame). Two large, inverted repeats (~7 kb each) with 100% identity were also identified, each harbouring a single copy of the *atp9* and *trnR* genes (Figure 3B). These repeats are located between positions 12,815–20,058 bp and 30,660–37,903 bp and are supported by long PacBio reads spanning the entire region, discarding assembly artifacts (Supplementary Figure S2).

### 3.5. Protein-Coding Genes in the Mitochondrial Genome

The total length of the 19 PCGs is 19,755 bp, accounting for 41.48% of the entire mitochondrial sequence of *W. anomalus.* Of these, 16 correspond to the core mitochondrial genes, representing 15 distinct genes (*atp6*, *atp8*, *atp9*, *cob*, *cox1*, *cox2*, *cox3*, *nad1*, *nad2*, *nad3*, *nad4*, *nad4l*, *nad5*, *nad6*, and *rps3*), with *atp9* present in two copies.

The remaining three genes code for proteins exhibiting similarities to the LD homing endonucleases (HEs). Two of these were identified by both MITOS2 [50] and MFannot [51] and are located within the *rrnL* and *cox1* genes. The third ORF, located within an intron of the *cob* gene, was predicted only by MITOS2 (Table 2). However, this intronic ORF is considerably long (>900 bp) and contains a well-conserved LD motif, suggesting that it is a true intron-encoded endonuclease. MITOS2 also predicted an additional short ORF (~140 bp) containing part of the LD motif. However, BLAST analysis showed strong similarity (E-values < 4 × 10^−93^) to *nad1* sequences from multiple fungal species. Consistent with this, MFannot re-annotation placed this sequence within the *nad1* coding region, and no Pfam domains associated with LD motifs were detected. We therefore annotated this region as part of the *nad1* gene.

Most PCGs start with the canonical translation initiation codon ATG, except for the LD-encoding genes, which are initiated with ATA. Two genes (*cox2* and *rrnL*) use TAG as the stop codon, while the remaining genes use TAA. Codon usage analysis indicated that the most frequently used codons were TTA (Leu), ATA (Met), AAT (Asn), TAT (Tyr), ATT (Ile), and TTT (Phe) with 718, 570, 471, 401, 311, and 230 counts, respectively. In contrast, the least commonly used codons were CGG (Arg), AGG (Arg), and CGC (Arg), each occurring once. Moreover, Relative Synonymous Codon Usage (RSCU) values revealed that ACT (4.05, Thr), AGA (3.44, Arg), and ACA (2.54, Thr) were used at disproportionately high frequencies relative to synonymous alternatives (Supplementary Table S6).

### 3.6. tRNA and rRNA Genes

**Figure 4:**
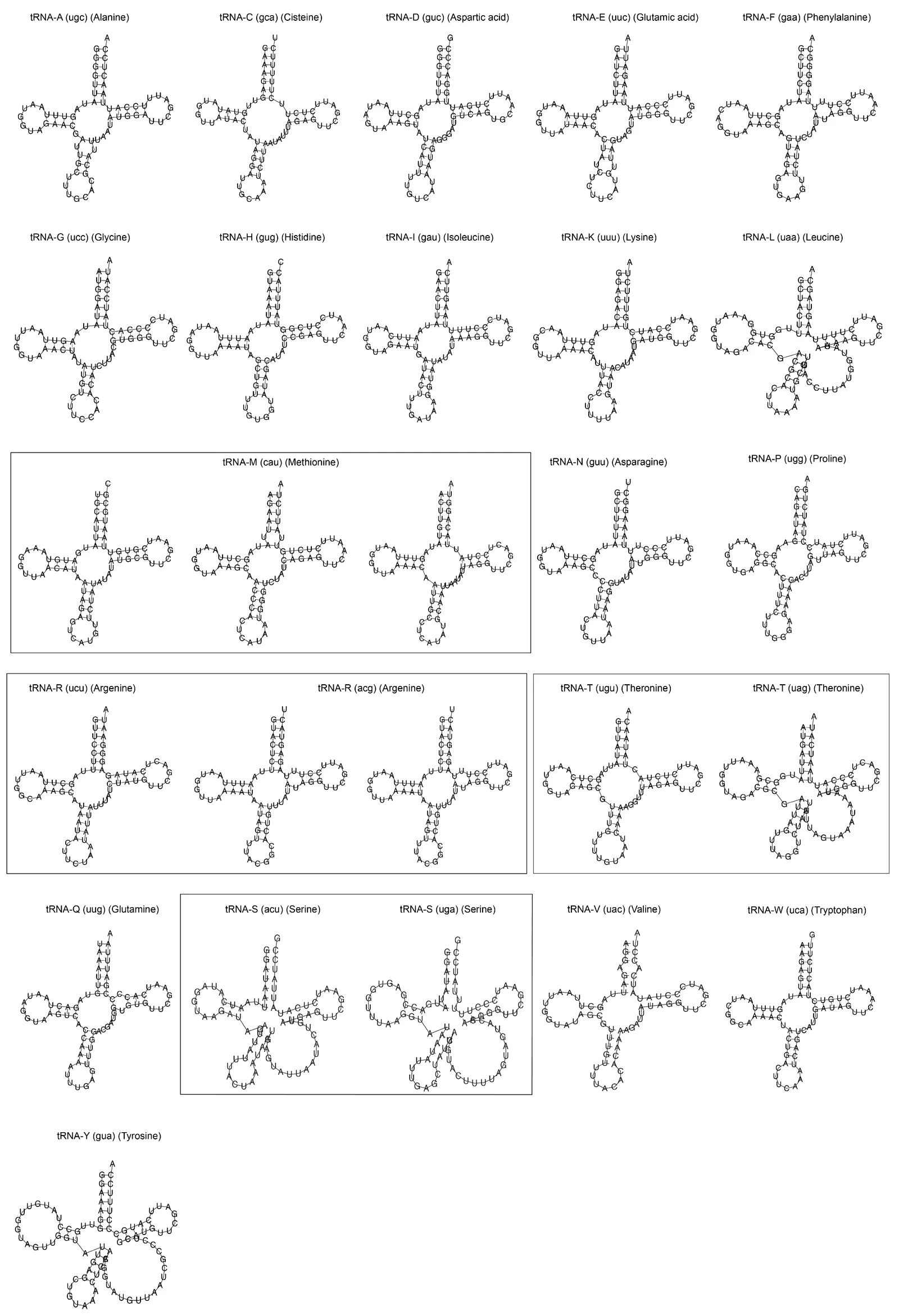
Secondary structure of 26 mt-tRNAs of *W. anomalus.*

The *W. anomalus* mitochondrial sequence contains a total of 26 tRNA genes with a total length of 1942 bp, accounting for 4.08% of the entire mitochondrial genome. All of the tRNAs exhibit the characteristic clover-leaf structure, and their average length ranged from 71 bp (*trnM*, *trnA*, and *trnI*) to 90 bp (*trnY*), which was mainly due to the variable arm (Figure 4). There is one tRNA for each amino acid with additional isoforms for the amino acids Methionine (Met), Arginine (Arg), Serine (Ser), and Threonine (Thr) (Table 2). The mitochondrial genome harbours three copies of the *trnM* (CAT) and *trnR* genes (two copies recognizing ACG, and one copy recognizing TCT), and two copies of the *trnS* (*trnS1* [ACT] and *trnS2* [TGA]) and the *trnT* genes (*trnT* [TAG], and *trnT* [TGT]).

Furthermore, the mitochondrial genome contains two rRNA genes, *rrnS* (small subunit ribosomal RNA) and *rrnL* (large subunit ribosomal RNA) with lengths of 1822 bp and 5083 bp, respectively. The *rrnS* was located between the *trnM* and the *trnV* genes (Figure 3), while the *rrnL* gene contains an LD-coding endonuclease (as mentioned above). While MFannot did not identify *rrnL*, initial annotation with MITOS2 predicted the gene as three separate fragments, misidentifying the region (45,192–45,272 bp) as part of an intron. This missing annotation was corrected through BLAST analysis against the *rrnL* sequences from *Wickerhamomyces mucosus* (KC993197.1) and *Saccharomyces cerevisiae* (OP414676.1 and KP712803.1), which identified three exons (Table 1). Infernal’s bacterial LSU rRNA model (RF02541) further confirmed conserved rRNA domains within exon 1 (44,838–44,897 bp, E-value 8.8 × 10^−6^) and exon 2 (45,194–45,270 bp, E-value 1 × 10^−18^). The final *rrnL* structure comprises three exons (41,875–44,895, 45,192–45,272, and 46,733–46,977, Table 2) separated by two intronic regions, one of which harbours the LD ORF.

### 3.7. Introns and Mobile and Repetitive Elements

Two group I introns hosting LD endonucleases were identified and classified into the group IA3 (positions 45,311 to 46,717, 1407 bp) within *rrnL*, and the group ID (25,041–26,456, 1415 bp) within *cox1*. Additionally, RNAweasel and Infernal (group I introns model, RF00028) [53] annotated a short 218 bp region as a group IA intron (44,913–45,130) between *rrnL*’s first and second exons, further supported by exonic alignments described in the previous section.

Both MITOS2 annotation and PFAM domain signature identified an LD-homing endonuclease domain within the *cob* gene. However, neither RNAweasel nor Infernal identifies a group I/II intron structure surrounding this ORF. We then aligned the *cob* gene sequence with intronless *cob* orthologs (*Kluyveromyces lactis*, *Wickerhamomyces canadensis*, and *Nakaseomyces glabratus*) and the intron-rich *S. cerevisiae cob* gene. The LD-HE region showed weak homology with the intronic regions bl1 and bl2 of *S. cerevisiae* (Supplementary Figure S3) but was absent in intronless species (Supplementary Figure S4). These results suggest that the LD ORF is not part of a functional group I intron and may represent either an intron degeneration event that retained the endonuclease ORF, or an intronless homing event.

Tandem repeat analysis identified 77 repeats, ranging from 31 bp (44,946–44,976, 10 copies of 3 bp) to 607 bp (9615–10,221, one copy of 543 bp), with 27 repeats having short units (2–15 bp, up to 74 copies) (Supplementary Table S7). Most repeats were located in intergenic regions (Supplementary Figure S5). RepeatMasker [55] identified 39 AT-rich simple sequence repeats (SSRs), with motifs such as (ATA)n, (TAA)n, and (TAT)n. Overlap analysis showed that 25 SSRs partially coincide with tandem repeats. Lastly, no transposable elements were found.

### 3.8. Comparative Mitochondrial Genome Analysis

**Figure 5:**
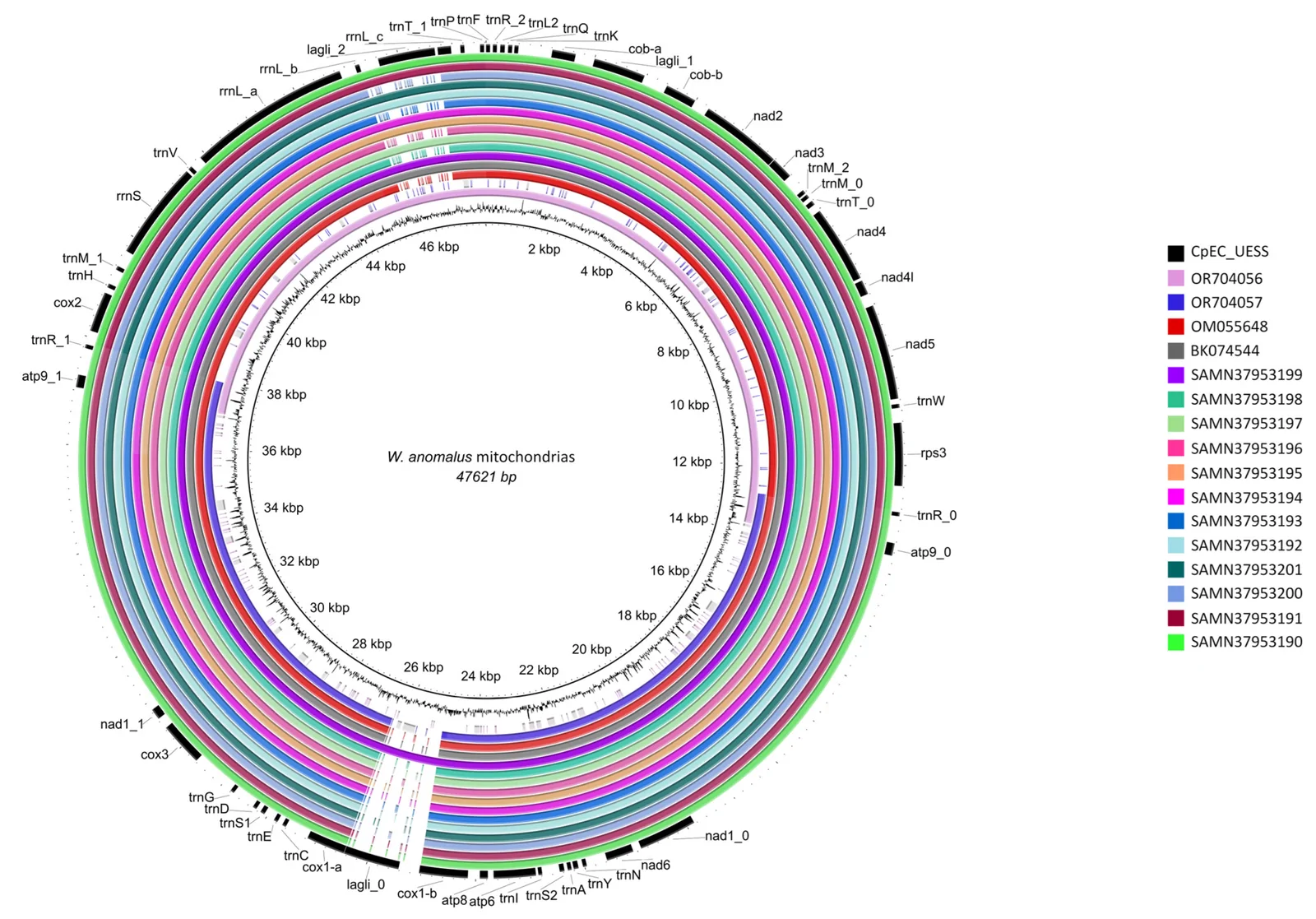
Comparison of mitochondrial genomes of *W. anomalus* clinical isolates. Circular BRIG-style alignment of 15 mitochondrial genome sequences (12 clinical isolates assembled in this study, the publicly available BK074544, OMO55648, and the partial sequences OR704056 and OR704057) against the CpEC_UEES reference (shown in black). Gene annotations are shown as black blocks on the outer ring. Coloured rings represent individual isolates as described in the legend; gaps indicate regions with no alignment to the reference.

*De novo* assembly of 12 additional clinical isolates of *W. anomalus* resulted in complete circular mitochondrial genomes of similar size and GC content to CpEC_UEES (Table 3). These 12 newly assembled clinical mitochondrial genomes were compared with the Ecuadorian isolate, and the publicly available sequences BK074544 (the mitochondrial genome of the clinical isolate SAMN37953190, which was reassembled in this study and included in the comparison), OR704056, OR704057, and OM055648 (the latter three labelled as unverified in NCBI). The sequences OR704056 and OR704057 were found to represent partial mitochondrial genome sequences rather than complete assemblies (Figure 5). The two sequences share approximately 0.9 kb of identical sequence at each of their termini and together reconstruct the complete circular mitochondria. These sequences were excluded from comparisons of genome size and GC content.

Overall, the mitochondrial genomes were highly conserved in size, gene content and synteny. The principal structural difference was the presence/absence of the LAGLIDADG (LD) homing endonuclease ORFs associated with the *cox1* and *rrnL* genes. The LD region within *rrnL* was highly variable among the clinical isolates, being present in 10 of 15 mitochondrial genomes (Figure 5). In contrast, the LD region associated with the *cox1* gene was found in only two isolates (CpEC_UEES and SAMN37953199).

### 3.9. Resistance and Virulence Factors

**Figure 6:**
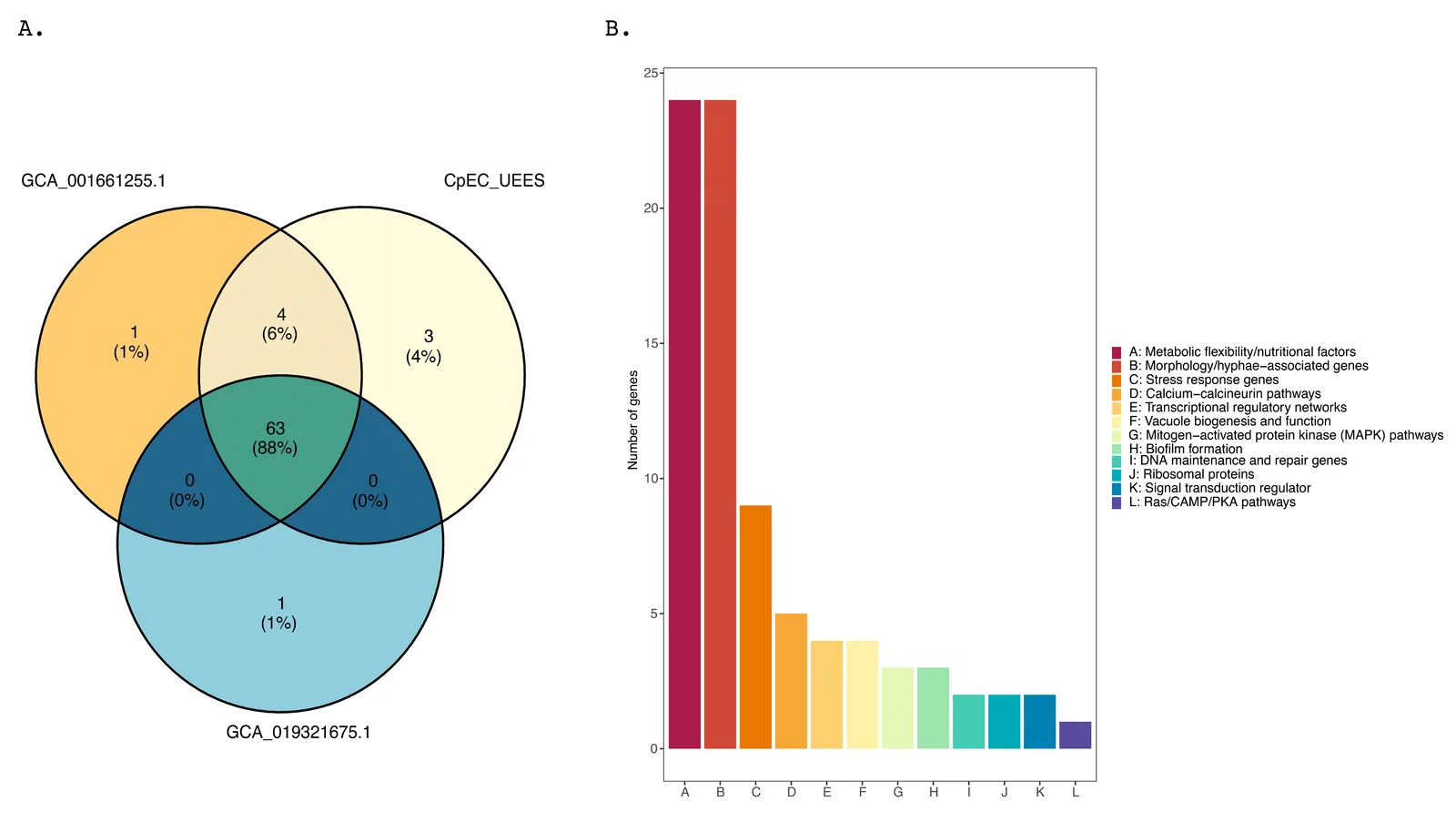
Distribution of pathogenic and virulence factors from CpEC_UEES, GCA_001661255.1, and GCA_019321675.1 genomes. (**A**). Venn diagram showing unique and shared virulence-associated genes in CpEC_UEES and reference genomes. (**B**). Mechanism-based classification and distribution of virulence factors found in the CpEC_UEES genome.

We identified a total of 70 virulence-associated proteins in the CpEC_UEES genome, 64 genes in the chromosome level genome available in the NCBI database (KG16_13) and 68 genes in the annotated reference genome (NRRL Y-366-8), which despite being an incomplete assembly, remains the only functionally annotated version (Figure 6A, Supplementary Table S8). Comparing these results with each other, we found a high number of virulence-associated genes shared among the reference genomes and the CpEC_UEES genome (63 proteins corresponding to 87.5%). We classified the determinant pathogenic and virulence factors into 11 groups based on their main mechanism and/or signalling pathways: calcium-calcineurin pathways, DNA maintenance and repair genes, metabolism flexibility/nutritional factors, mitogenic-activated protein kinase (MAPK) pathways, morphology/hyphae-associated genes, Ras/CAMP/PKA pathways, ribosomal proteins, signal transduction regulators, stress response genes, transcriptional regulatory networks, and vacuole biogenesis and function (Figure 6B). Most proteins (*n* = 22) were involved in metabolic pathways, followed by morphology/hyphae-associated genes (*n* = 19), and stress response genes (*n* = 9).

Results of the MaRDy analysis [63] yielded no matching results. This finding is consistent with the phenotypic observations, wherein we observed susceptibility to the antifungals tested.

### 3.10. Prediction of Adhesin Function of Annotated Proteins

Adhesion to host cells is a key early step in fungal infections, mediated mainly by cell wall adhesins. In *W. anomalus*, adhesion has been proposed as an infection mechanism since strains of this species show high levels of adherence and cytotoxicity to epithelial cells [73]. We therefore looked for adhesin-like proteins in the CpEC_UEES predicted-proteome. A total of 35 protein sequences were predicted as adhesins, from which 14 showed a query coverage and percentage of identity greater than 20% to known proteins from related fungal species (Table 4). Following the mechanism-based classification (see above), these sequences belong to three main groups: morphology/hyphae-associated proteins (*n* = 8), metabolic flexibility/nutritional factors (*n* = 2), biofilm formation (*n* = 3), and one calcium-calcineurin pathways protein (Supplementary Table S9).

### 3.11. Phylogenetic Relationship

**Figure 7:**
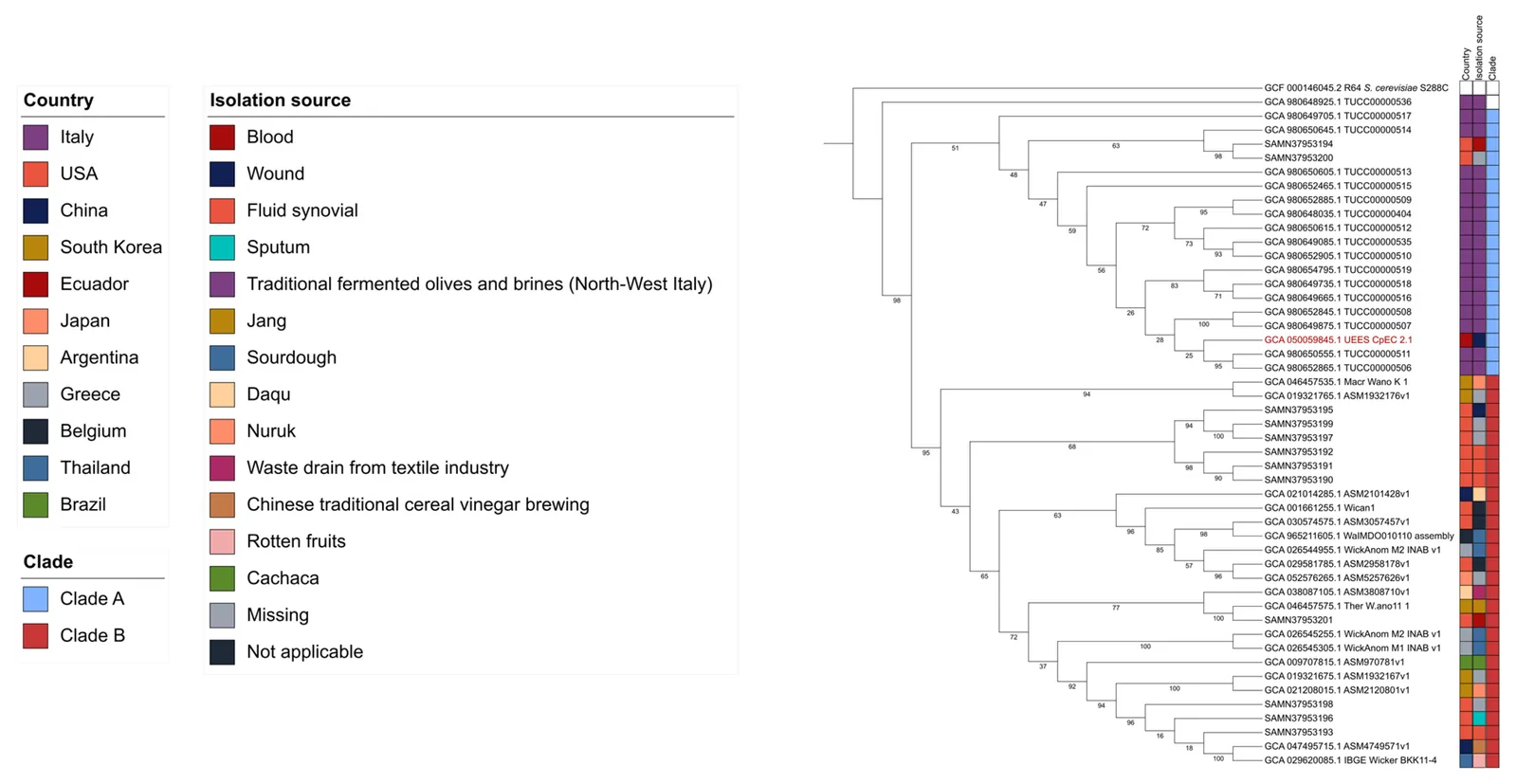
UFCG core-gene phylogeny of *W. anomalus* isolates. The maximum-likelihood phylogeny was inferred with UFCG from a concatenated alignment of 55 core genes across 49 genome assemblies, rooted with *S. cerevisiae* S288C (GCF_000146045.2) as outgroup. Branch support was assessed with 1000 ultrafast bootstrap replicates. The tree was visualized in iTOL [69]. Coloured bars adjacent to tip labels indicate country of origin and isolation source, as defined in the legend.

We compared 48 assemblies of *W. anomalus* and reconstructed their phylogeny with UFCG using *S. cerevisiae* S288C as an outgroup (Figure 7). The phylogenetic tree split into two major clades (bootstrap = 98). One clade comprised the Ecuadorian isolate CpEC_UEES, Italian isolates from fermented olives and brines and two additional clinical samples (SAMN37953194 and SAMN37953200). The second clade contained the remaining assemblies, including ten clinical isolates. Importantly, the clinical isolates analysed here do not form a single monophyletic group but rather were distributed across the tree.

However, three well-supported subclusters consistently recurred among the clinical isolates within the second clade. The first subcluster included SAMN37953195/197/199 from wound and two from undetermined sources, respectively. The second subcluster is formed by the samples SAMN37953190/191/192, all sourced from synovial fluid. The last group, SAMN37953193/196/198, clustered with two samples from China and Thailand, respectively. Lastly, the remaining clinical isolate, SAMN37953201, clustered with the industrial sample Ther W.ano11 (bootstrap = 100) rather than with other clinical strains.

Isolates from other countries or other fermentation contexts (jang, sourdough, daqu or nuruk), were randomly distributed within the second major cluster, rather than grouping by source. Nonetheless, some geographic structure was evident in two South Korean sister pairs (bootstrap = 94 and 100).

## 4. Discussion

Previous studies have consistently reported an increasing incidence of infections caused by uncommon agents of candidemia. Among these agents, *W. anomalus* has been classified as an emergent pathogenic yeast, which is increasingly reported as a source of infection in clinical settings, primarily affecting neonates and infants [3,7]. Although it has been associated with poor clinical outcomes, therapeutic failures, and clonal outbreaks, our understanding of how its genome structure relates to its pathogenesis and virulence is limited. The present study reports the first nuclear and complete mitochondrial genome of a clinical isolate of *W. anomalus* from a wound secretion. Additionally, we assembled the genomes of 12 clinical isolates from raw sequencing reads (SRA) available at NCBI for mitochondrial and core-gene phylogenetic analysis. All other *W. anomalus* assemblies available at NCBI have an environmental origin. To reach these results, a hybrid read-based assembly approach complemented with short read polishing was chosen to combine the advantages of the Illumina short reads and PacBio long reads sequencing methods. The final draft genome consists of 20 contigs and 10 scaffolds, with an estimated genome size of 13.96 Mb, 34.51% GC, and 0.03% Ns. The contig N50 and scaffold N50 were 1.28 Mb and 2.1 Mb, respectively. We also identified a total of 6324 protein-coding genes, 173 tRNAs, and 11 truncated transposable elements. Accordingly, the reference genome of *W. anomalus* presents similar genome size (14.1 Mb), GC percentage (34.5%), contig N50 (185.3 kb), scaffold N50 (2.2 Mb), and protein-coding genes (6421). Our results show a more contiguous and higher-quality genome compared to the reference genome, despite one of the 10 scaffolds remaining unplaced. We further report on the first published complete mitochondrial genome assembly of *W. anomalus.*

### 4.1. Characteristics of W. anomalus Mitochondrial Genome

The mitochondrial genome of *W. anomalus* exhibits a total size of 47,621 bp and a GC content of 20.8%, which is consistent with previously reported yeast mitochondrial genomes [74]. Core mitochondrial genes encoding components of oxidative phosphorylation (*atp6*, *atp8*, *atp9*, *nad1–6*, *nad4l*, *cox1–3*, and *cob*) were present. While these genes are conserved across fungal species, some fungal species may lack some core mitochondrial genes due to their migration to the nuclear genome during evolution. For instance, *Wickerhamomyces pijperi*, a close relative of *W. anomalus*, has independently lost all Complex I genes [74]. No genes associated with pathogenicity were detected, which is in agreement with the mitochondrial genomes of non-pathogenic fungi that focus more on fundamental metabolic activities, such as ATP production and biosynthesis, without influencing virulence or stress response.

A striking feature of the *W. anomalus* mitochondrial genome is the presence of two large, inverted repeats (~7 kb each, Figure 3B). LIRs are common in chloroplast genomes but are rare in mitochondrial genomes. In fungi, LIRs have been identified in a limited number of genera, including *Candida*, *Saccharomyces*, *Hanseniaspora*, *Agaricus*, *Termitomyces*, and *Malassezia* [75,76]. These repeats function as recombination hotspots in *C. albicans* and may contribute to replication, structural rearrangements, and genome stability [77]. In *Malassezia*, LIR formation has been linked to the duplication and inversion of the ancestral syntenic unit *trnR-trnL-atp9*, also found in *Ustilago maydis* and *Pseudozyma* spp. [75]. Interestingly, the LIRs of *W. anomalus* exhibit a similar inverted *trnR-atp9* arrangement, but lack *trnL*, suggesting that a similar recombination-driven mechanism underlies their formation. The two inverted repeats are identical to each other, indicating ongoing homologous recombination.

In fungi, the *atp9* gene is either absent from the mitochondrial genome, present as a single copy, or, in rare cases, duplicated [78] or within LIRs [75]. Other key mitochondrial genes, including *atp6*, *nad2*, *nad4*, and *rrnl*, have been found within LIRs in certain fungi [75,76]. Whether such inverted duplications confer a dosage effect that enhances fitness remains uncertain given that mitochondrial gene functions are highly conserved and most fungi lack LIRs or duplicated genes in their mitochondrial genomes. In *W. anomalus*, the presence of two *atp9* copies within the LIRs may provide a fitness advantage by ensuring stable ATP production during infection. However, further studies are needed to confirm the dosage effect or fitness advantage.

Fungal mitochondrial genomes are highly diverse, mainly due to the presence of mobile genetic elements (group I and group II introns) and repetitive sequences [74]. In this study, we identified three LAGLIDADG-type HEGs, two of which are located within group I introns: one in a group ID intron in the *cox1* gene and one in a group IA3 intron in the *rrnL* gene. Most noteworthy is the presence of an LD ORF in the *cob* gene without an identifiable intronic sequence, as tools like Infernal and RNAweasel failed to detect neighbouring intronic regions. This might be due to sequence degeneration reducing similarity to conserved intron models.

Notably, 48.9% (23,297 bp) of the mitochondrial genome corresponds to non-coding content, primarily consisting of intergenic regions, intronic sequences (excluding LD ORFs), and repetitive elements. This unannotated fraction is within the typical range for yeast mitochondrial genomes in the Saccharomycetales order, such as *Saccharomyces* and *Candida*, where non-coding content can vary from 10% to over 50% [79]. Of the non-coding region, 6.85% corresponds to intronic sequences excluding the LD ORFs, and RepeatMasker and equicktandem analysis revealed that 27.06% (6304 bp) of the unannotated area consisted of repetitive elements, including tandem repeats and SSRs. These repeats, together with intron composition, may contribute to structural variation and plasticity.

The CpEC_UEES mitochondrial genome was compared with 15 additional complete mitochondrial assemblies of which two are publicly available in NCBI. The mitochondrial genome size, gene content and synteny are highly conserved within this species. The principal structural variation among the isolates was the presence or absence of the two LD ORFs within the *rrnL* and *cox1* genes. The *rrnL*–associated element was highly variable, present in 10 of 15 isolates, while the *cox1*-associated LD ORF was detected only in two clinical isolates (CpEC_UEES and SAMN37953199).

Whether such variations impact the pathogenicity or adaptation of these clinical isolates remains uncertain, given the high diversity observed in fungal mitochondrial genomes even at the intraspecies level [80], consistent with the variable presence of LD ORFs among the 14 clinical and environmental isolates. Nonetheless, mitochondrial genome variations, including intron content, have been associated with differences in virulence, pathogenicity, and drug resistance in several fungal species [24]. Therefore, a regulatory role of these elements in strain fitness and virulence cannot be excluded, and association studies between mitochondrial genomes and phenotypic data would be required.

### 4.2. Core-Genome Phylogeny of Clinical W. anomalus Isolates Reveals a Non Monophyletic Lineage

In the clade A, the clinical isolates from Ecuador and the U.S.A are phylogenetically related to isolates from Italian fermented food. Clade B, on the other hand, consists of widely geographically distributed isolates from multiple industrial and fermented sources. This suggests that both clades are not restricted to particular geographic regions or ecological niches.

This wide distribution pattern argues against the existence of a dedicated, virulent clinical sublineage, and instead supports an opportunistic and sporadic model of infection. This is consistent with the comparable pathogenicity previously reported for clinical and environmental *W. anomalus* strains in *G. mellonella* [73]. Given that *W. anomalus* is widely used in fermentation industries, the absence of a clear phylogenetic boundary between clinical and industrial strains suggests that virulence potential cannot be predicted from taxonomic or geographic origin alone and supports continued monitoring of strains used in industrial settings, particularly given this species’ capacity for opportunistic infections. Broader sampling of both clinical and environmental samples will be needed to determine whether specific genomic features are associated with clinical origin or pathogenic potential.

### 4.3. Virulence-Associated Factors Are Present in Clinical and Ecological Wickerhamomyces anomalus Genomes

As an opportunistic pathogen, *W. anomalus* demonstrates a capacity to colonise tissues and initiate infections in various hosts. A previous study reported its ability to induce lethal outcomes in the invertebrate model *Galleria mellonella*, highlighting its pathogenicity [73]. To elucidate factors underlying its pathogenic potential, we conducted a comparative analysis between the predicted protein sequences and known virulence traits of different *Candida* species. Pathogen–host interactions revealed 70 proteins with high similarity to those in the database, of which 63 were conserved across all examined genomes, reflecting the substantial genomic congruence observed. Consistent with this, Perez-Traves et al. (2021) found similar levels of pathogenicity in *G. mellonella* larvae among *W. anomalus* strains regardless of their origin (clinical or environmental) [73]. Together, these observations suggest that the pathogenic potential of *W. anomalus* is not determined by gene content alone. Instead, differences in virulence among strains may result from variation in gene regulation, including transcriptional responses to host conditions and differences in protein abundance. In *C. albicans*, for instance, the contribution of *PHR1* and *PHR2* to virulence depends on their pH-regulated expression in different host niches [81]. In *W. anomalus* itself, stress tolerance involves transcriptional and metabolic reprogramming, including the induction of antioxidant enzymes [11]. Comparative transcriptomic and proteomic analyses of clinical and environmental isolates might elucidate regulatory mechanisms underlying the pathogenic potential of this yeast.

We also identified adhesion-associated proteins that play a critical role in the infection process of pathogenic fungi. Based on the presence of a GPI-anchor domain, signal peptide sequence, and homology to known fungal adhesion proteins, we found a total of 12 unique adhesin-like GPI-anchored proteins orthologous to those present in *Candida albicans*, *Candida orthopsilosis*, and *Cyberlindnera fabianii*, indicating a potential predisposition to virulence and biofilm formation.

We combined and classified these determinant pathogenic and virulence factors (83 unique proteins) into 12 groups based on their main mechanisms, wherein metabolic flexibility/nutritional factors, morphology/hyphae-associated genes, and stress response genes were the most enriched groups with 68.7% (57 proteins) of the predicted protein sequences.

### 4.4. W. anomalus Contains Metabolic Flexibility/Nutritional Factors That Might Facilitate Its Adaptation to Host Environments

*W. anomalus* is considered a robust organism due to its ability to grow under various carbon and nitrogen sources, as well as extreme environmental stress conditions such as low pH, high osmotic pressure, and anaerobic conditions [11,12,13]. Such remarkable metabolic versatility may play a pivotal role in the fungus’s virulence by facilitating adept nutrient assimilation and its adaptation to host microenvironments. Indeed, previous research on opportunistic fungi such as *Candida albicans*, *Aspergillus fumigatus*, and *Cryptococcus neoformans* highlights their metabolic flexibility as an integral part of their pathogenicity [82,83,84].

As expected, we identified several genes that are part of different metabolic pathways that impact *Candida* virulence. Among these, there are the glyoxylate cycle genes *ICL1* (Isocitrate lyase) and *MLS1* (Malate synthase), which are required to utilize alternative carbon sources amidst nutrient scarcity [85,86]. Notably, *ICL1* is also required for prolonged survival following macrophage engulfment of *C. albicans* and *C. glabrata* [85,87].

We also found homologous sequences of the two main enzymes of the trehalose biosynthesis pathway, Tps1 (trehalose-6-phosphate synthase) and Tps2 (Trehalose-6-phosphate phosphatase), as well as the neutral trehalase enzymes Ntc1, Nth1, and Nt2. The synthesis and accumulation of the nonreducing disaccharide trehalose confer protection under stress conditions to several organisms, including *W. anomalus* [88,89]. Accordingly, diminished tolerance to oxidative stress, decreased susceptibility to macrophage-mediated clearance, and lower infectivity are observed in *Candida* mutants unable to synthesise this disaccharide [90,91,92,93].

The metabolic versatility of *W. anomalus* is further demonstrated by the presence of essential transporters required for nutrient uptake and accumulation, such as Dur1_2 (urea transporter), Fth2 (iron transporter), Vht1 (biotin transporter), Ftr1 (high-affinity iron permease), and Gap1 (general amino acid permease).

### 4.5. Morphology/Hyphae-Associated Genes

Fungi exhibit morphological plasticity as a means to adapt to changing environmental conditions, including variations in nutrient availability and mating factors. The composition and organisation of the cell wall are tightly linked to the morphology of the cells, influencing their shape, size, and growth pattern [94]. In this study, we identified several genes that are orthologs to the cell wall-associated genes *UTR2*, *PHR1*, and *PHR2*. *UTR2*, which encodes a transglycosidase that links 1,3-β-glucan to chitin, is involved in response mechanisms to cell wall-degrading enzymes [94,95]. Accordingly, Δ*utr2* mutants of *C. albicans* showed increased sensitivity to echinocandin treatment, altered morphology, reduced cell adhesion, and attenuated virulence in a mouse model of systemic candidiasis [94,95].

*PHR1* and *PHR2* are responsible for encoding glycosylphosphatidylinositol-anchored cell surface proteins with morphological functions [96]. Although deletion of *PHR1* and *PHR2* from *C. albicans* and their respective homologous genes in *S. cerevisiae* and *C. glabrata* results in altered cell wall biogenesis, reduced adhesive properties, and morphological defects [97,98], these phenotypes are dependent on ambient pH in *C. albicans* and *C. dubliniensis* [96,99]. In contrast to Δ*phr1* cells, which are morphologically abnormal at alkaline pH (greater than 6), Δ*phr2* mutants display morphological defects at acidic pH [100]. Accordingly, the former mutants are avirulent in a mouse model of systemic candidiasis but display unaltered virulence in rat models of vaginal infection [81,96]. The inverse phenotype is observed in Δ*phr2* mutants.

*W. anomalus* typically grows as yeast but can adopt pseudohyphal structures [73,101]. Nonetheless, pseudohyphae appeared to have a minimal influence on the virulence-associated mechanisms of cytotoxicity and damage potential [73]. Adhesion, on the other hand, was proposed as a plausible mechanism of infection of this yeast, as *W. anomalus* strains showed high levels of adherence and cytotoxicity to epithelial cells [73]. The role of *UTR2*, *PHR1*, and *PHR2* in the morphology and adhesion across evolutionarily distant fungi suggests a conservation of their functions. Thus, the homologue genes of *UTR2*, *PHR1*, and *PHR2* might have similar roles in the adhesion properties of *W. anomalus*; however, their impact on the virulence of the yeast should be further studied.

### 4.6. Stress-Response Genes

The draft genome of *W. anomalus* reveals the presence of several genes that code for superoxide dismutases (SODs) and catalases (CATs). These enzymes are considered the first line of defence against oxidative stress in eukaryotic organisms. In the case of *W. anomalus*, a high activity of *SOD* genes was observed following ethanol stress [11]. However, these were not sufficient to ameliorate growth inhibition and damage to cell membrane integrity. Interestingly, while studies have not explored the impact of SOD expression in *W. anomalus* under other environmental factor stresses, research on other opportunistic fungi has linked SODs to their virulence. For instance, *C. albicans* Δ*sod1* mutants exhibited increased susceptibility to phagocytosis and superoxide radicals produced by the immune system, and attenuated virulence in a mouse model [102]. Similarly, SODs protect against the oxidative burst of the host immune response in *Paracoccidioides* spp., *Histoplasma capsulatum*, and *Beauveria bassiana*, suggesting that the functions of these enzymes might be conserved [103,104,105].

Although shown to be inefficient under ethanol stress, *W. anomalus* SOD and CAT enzymes might be adequate to confer protection against superoxide radicals in the context of immunosenescence. This is supported by observations that senescent neutrophils present impaired oxidative response and phagocytic defects [106,107]. Nonetheless, further research is required to establish the role of these enzymes in *W. anomalus* infection.

This study has several limitations. First, the studied clinical isolates originated from a limited number of countries and infection sites, thus the dataset may not capture the full genomic diversity of clinical *W. anomalus*. A larger number of samples of clinical isolates from diverse regions with standardised clinical metadata will be needed to confirm the observations presented in this study. Second, proteins associated with virulence were identified by homology and computational prediction. Their expression and biological functions were not evaluated experimentally in this clinical isolate. Nevertheless, the genomic sequence was supported by high sequencing depth, with Illumina reads providing an average depth of approximately 95× and more than 98% of reads mapping to the final assembly, supporting the reliability of the sequence-based predictions. Therefore, these proteins should be regarded as candidate virulence associated homologous proteins that require functional validation. Third, the thresholds applied to the PHI-base BLASTP search (E-value < 1 × 10^−6^, sequence identity > 60%, query coverage > 80% and hits restricted to *Candida* spp.) were deliberately stringent. As a result, more divergent homologues of known virulence factors, as well as virulence-associated proteins characterised in other fungal genera, may have been missed. The set of candidate virulence-associated proteins reported here should therefore be considered conservative and may underestimate the virulence repertoire of this isolate.

## 5. Conclusions

Our study provides the first hybrid nuclear and complete mitochondrial genome overview of a clinical isolate of *W. anomalus* from Ecuador, together with complete mitochondrial assemblies of 12 clinical isolates from the U.S.A. The mitochondrial genome contained unusual structural features, including two large, inverted repeats carrying duplicated atp9 copies, and comparative analysis showed high conservation in size, gene content, and synteny across isolates, with structural variation in the presence or absence of LD endonuclease ORFs. Phylogenetic analysis further showed that clinical isolates do not form a single lineage but are instead interspersed with strains from food fermentation and industry, consistent with an opportunistic and sporadic mode of infection. In line with this, most candidate virulence-associated homologs identified in CpEC_UEES were shared with environmental genomes. This report is a step forward toward the initiation of genomic studies of this robust fungus, which is responsible for several outbreaks around the world and has been associated with poor clinical outcomes. Although the isolate under investigation did not present resistance to the tested antifungals, reported resistance to flucytosine and triazoles in other isolates could pose a concern in clinical settings in the future. Given the absence of clear phylogenetic separation between clinical and environmental samples and its dual identity as both an opportunistic pathogen and an industrially valuable organism, continued genomic surveillance is essential to detect changes that may enhance its virulence or environmental adaptability.

## Figures and Tables

**Table 1:** Summary information of the contig-level assemblies generated with the five different assemblers used. All assemblies were polished three times before comparison.

	Wengan	MaSuRCA	Flye	SMARTdenovo	Canu- SMARTdenovo
Contig Numbers	342	20	52	30	29
Total genome size (bp)	15,641,129	13,991,713	14,001,624	14,097,927	14,004,920
GC content (%)	34.63	34.51	34.69	34.53	34.56
N50 (bp)	74,133	1,283,267	1,422,051	891,120	960,118
N75 (bp)	39,304	1,112,314	566,893	583,755	576,198
Largest Contig	294,006	2,946,481	2,746,987	3,632,912	3,626,365
QV short reads	30.77	47.95	31.64	28.98	35.31
QV long reads	30.51	47.42	31.68	29.06	35.45
BUSCO score completeness	67%	98.1%	96.4%	88.7%	96.6%

**Table 2:** Annotation of the *W. anomalus* mitochondrial genome.

Genes	Orientation	Position	Function
*tRNA^Phe^*	(+)	1–73	Anticodon: GAA
*tRNA^Arg^*	(+)	125–197	Anticodon: UCU
*tRNA^Leu^*	(+)	261–342	Anticodon: UAA
*tRNA^Gln^*	(+)	405–478	Anticodon: UUG
*tRNA^Lys^*	(+)	520–592	Anticodon: UUU
*cob*	(+)	join(1213–1644, 3251–3984)	Apocytochrome b
*lagli* (3)	(+)	1999–2958	Maturase domain of enzymes promoting RNA splicing and intron homing
	(−)	25,420–26,448	
	(+)	45,632–46,678 *	
*nad2*	(+)	4269–5774	NADH dehydrogenase subunit 2
*nad3*	(+)	5788–6192	NADH dehydrogenase subunit 3
*tRNA^Met^* (3)	(+)	6544–6615,	Anticodon: CAU
		6650–6720,	
		39,334–39,406	
*tRNA^Thr^*	(+)	6797–6879	Anticodon: UAG
*nad4*	(+)	7029–8459	NADH dehydrogenase subunit 4
*nad4l*	(+)	8514–8780	NADH dehydrogenase subunit 4L
*nad5*	(+)	9009–10,769	NADH dehydrogenase subunit 5
*tRNA^Trp^*	(+)	10,868–10,939	Anticodon: UCA
*rps3*	(+)	11,212–12,369	Small ribosomal subunit protein uS3
*tRNA^Arg^* (2)	(−)	12,851–12,922	Anticodon: ACG
	(+)	37,796–37,867	
*atp9* (2)	(−)	13,423–13,653	ATP synthase F_0_ subunit 9
	(+)	37,065–37,295	
*nad1*	(−)	19,849–20,901	NADH dehydrogenase subunit 1
*nad6*	(−)	21,073–21,576	NADH dehydrogenase subunit 6
*tRNA^Asn^*	(−)	21,958–22,029	Anticodon: GUU
*tRNA^Tyr^*	(−)	22,114–22,203	Anticodon: GUA
*tRNA^Ala^*	(−)	22,242–22,312	Anticodon: UGC
*tRNA-S2^Ser^*	(−)	22,375–22,461	Anticodon: UGA
*tRNA^Ile^*	(−)	22,785–22,855	Anticodon: GAU
*atp6*	(−)	22,900–23,673	ATP synthase F_0_ subunit 6
*atp8*	(−)	23,780–23,926	ATP synthase F_0_ subunit 8
*cox1*	(−)	join(24,145–25,038, 26,455–27,168)	Cytochrome c oxidase subunit I
*tRNA^Cys^*	(−)	27,615–27,686	Anticodon: GCA
*tRNA^Glu^*	(−)	27,791–27,862	Anticodon: UUC
*tRNA-S1^Ser^*	(−)	28,067–28,148	Anticodon: ACU
*tRNA^Asp^*	(−)	28,241–28,312	Anticodon: GUC
*tRNA^Gly^*	(−)	28,743–28,814	Anticodon: UCC
*cox3*	(−)	29,638–30,447	Cytochrome c oxidase subunit III
*cox2*	(+)	38,127–38,867	Cytochrome c oxidase subunit II
*tRNA^His^*	(+)	38,975–39,047	Anticodon: GUG
*rrnS*	(+)	39,723–41,544	15S rRNA gene
*tRNA^Val^*	(+)	41,594–41,666	Anticodon: UAC
*rrnL*	(+)	join(41,895–44,895, 45,192–45,272, 46,733–46,977)	21S rRNA gene
*tRNA^Thr^*	(+)	47,150–47,222	Anticodon: UGU
*tRNA^Pro^*	(+)	47,512–47,583	Anticodon: UGG

* ORF is within the *rrnL*; this will be denoted as lagli-rrnL.

**Table 3:** Characteristics of available mitochondrial sequences.

Assembly	Size (bp)	GC Content (%)	kmer-Coverage
SAMN37953199	48,575	20.93	1134.2
SAMN37953198	45,412	20.71	825.5
SAMN37953197	46,746	20.69	1000.4
SAMN37953196	45,412	20.71	1004.6
SAMN37953195	46,916	21.07	412.7
SAMN37953194	46,916	21.07	579.9
SAMN37953193	45,412	20.71	720.3
SAMN37953192	46,746	20.69	437.6
SAMN37953201	46,330	19.81	412.5
SAMN37953200	45,302	20.56	677.8
SAMN37953191	46,746	20.69	369.8
SAMN37953190	46,746	20.69	340.8
OM055648	45,302	20.55	NA
BK074544	46,746	20.69	NA

**Table 4:** Hypothetical orthologous proteins of the predicted adhesin-like protein sequences.

Name of Proteins	Hypothetical Orthologous Proteins				
	Gene	Description	Species	Query Coverage	Per. Identity
Cpelliculosa_G0004250| XP_019041806.1	*CTSD*	Hypothetical aspartic protease	*Cyberlindnera fabianii*	85%	61.81%
Cpelliculosa_G0004470| XP_019041787.1	*ENG1*	Endo-1,3-beta-glucanase	*Candida albicans*	82%	31.52%
Cpelliculosa_G0005290| XP_019041706.1	*SAP4*	Secreted aspartyl proteinase 4	*Cyberlindnera fabianii*	37%	28.72%
Cpelliculosa_G0007900| XP_019041444.1	*UTR2*	Chitin transglycosylase	*Candida albicans*	81%	50.00%
Cpelliculosa_G0014410| XP_019040826.1	*ALS2*	Aglutinin-Like Sequence 2	*Candida albicans*	92%	25.94%
Cpelliculosa_G0025120| XP_019039815.1	*PGA59*	Predicted GPI-Anchored Protein Associated 59. Cell wall protein	*Candida orthopsilosis*	88%	48.87%
Cpelliculosa_G0045280| XP_019037675.1	*YWP1*	Yeast-form Wall Protein 1	*Cyberlindnera fabianii*	99%	44.26%
Cpelliculosa_G0045490	*FIG2*	Factor-Induced Gene 2	*Cyberlindnera fabianii*	81%	38.77%
Cpelliculosa_G0045790| XP_019037616.1	*PHR2*	pH-responsive protein 2	*Candida albicans*	88%	66.32%
Cpelliculosa_G0045800| XP_019037615.1	*PHR1*	pH-responsive protein 1	*Candida albicans*	80%	61.57%
Cpelliculosa_G0047350| XP_019037461.1	*MID1*	Stretch-activated cation channel	*Candida albicans*	74%	38.43%
Cpelliculosa_G0050730| XP_721564.1/YNR067C	*ENG1*	Endo-1,3-beta-glucanase	*Candida albicans*	89%	48.85%
Cpelliculosa_G0051480| XP_019037037.1	*PGA14*	Hydrophilin PGA14	*Candida albicans*	59%	50.00%
Cpelliculosa_G0060650| XP_019036399.1	*PHR1*	pH-responsive protein 1	*Candida albicans*	77%	49.14%

## Data Availability

Whole genome sequencing data have been deposited in the NCBI database under the BioSample accession number SAMN39985750.

## Supplementary Materials

The following supporting information can be downloaded at https://doi.org/10.6084/m9.figshare.33301593 (accessed on 26 September 2026). Figure S1. Whole genome synteny comparison across the de novo assemblies and the reference genome. A synteny plot compares the *W. anomalus* genome assembled with MaSuRCA and Canu-SMARTdenovo against the reference assembly GCA_019321675.1. Gray ribbons mark conserved collinear regions, while red ribbons denote inversions or rearranged genomic segments. The colour scale shows sequence identity from 78% to 100%. Figure S2. Long-read alignment validates the mitochondrial genome assembly. Long sequencing reads were mapped onto the assembled *W. anomalus* mitochondrial genome across a representative genomic segment. The upper panel shows coverage depth, and the lower panel shows the individual mapped reads. Uniform coverage and uninterrupted read alignments indicate the assembly’s accuracy and structural continuity. Figure S3. Multiple sequence alignment of mitochondrial genomes from four yeast species. Pairwise alignment of the *W. anomalus* mitochondrial genome with those of *C. glabrata*, *K. lactis*, and *W. canadensis.* Conserved nucleotide positions are shown in red, while polymorphic sites, insertions, and deletions are left unshaded. Figure S4. Enlarged view of the conserved mitochondrial region shown in Figure S3. Conserved nucleotide blocks are marked in red. Figure S5. Circular map of the *W. anomalus* mitochondrial genome. Circular representation of the 47,621-bp mitochondrial genome of *W. anomalus*. The outer ring shows protein-coding genes (blue arrows), transfer RNA genes (red), and ribosomal RNA genes (green). Internal rings indicate tandem repeats (purple) and simple sequence repeats (SSRs; orange). Table S1. Isolate resistance profile. Table S2. Summary of the contigs assembly features. Table S3. GO annotations. Table S4. KOG annotations. Table S5. KEGG annotations. Table S6. Codon usage frequency. Table S7. Mitochondrial repetitive elements. Table S8. Resistance and virulence factors (CpEC-UEES). Table S9. Predicted adhesin-like proteins.

## Abbreviations

BUSCO	Benchmarking Universal Single-Copy Orthologs
GO	Gene Ontology
KOG	euKaryotic Orthologous Groups
KEGG	Kyoto Encyclopedia of Genes and Genomes
RSCU	Relative Synonymous Codon Usage
ORF	open reading frame (marco de lectura abierto)
SSR	simple sequence repeat (repeticiones simples)
SNPs	single-nucleotide polymorphism
GPI	glycosylphosphatidylinositol
PCG	protein-coding gene (gen codificante de proteína)
tRNA	transfer RNA
rRNA	ribosomal RNA
rrnS	small subunit ribosomal RNA (15s)
rrnL	large subunit ribosomal RNA (21s)
HE	homing endonuclease
ccs	circular consensus sequencing
MIC	minimum inhibitory concentration
